# A retrospective study of estrogen in the pretreatment for medical management of early pregnancy loss and the inference from intrauterine adhesion

**DOI:** 10.1186/s40001-022-00767-z

**Published:** 2022-07-25

**Authors:** Chaoxia Cao, Qin Zhou, Zhuoying Hu, Chunmei Shu, Mingju Chen, Xiujun Yang

**Affiliations:** Department of Obstetrics and Gynecology, The First Affiliated Hospital of Chongqing Medical University, Chongqing, People’s Republic of China

**Keywords:** Mifepristone, Early pregnancy loss, Hysteroscopy, Intrauterine adhesion, Estradiol valerate

## Abstract

**Background:**

Estrogen has been usually used in clinic for medical pretreatment of early pregnancy loss. There was little reported the effect of estrogen combined with prostaglandin analogs in the medical management of early pregnancy loss. This retrospective study aimed to evaluate the efficacy of estrogen pretreatment for medical management of early pregnancy loss and explore the confounding factor of intrauterine adhesion (IUA) on the outcome of medical management.

**Methods:**

A total of 226 early pregnancy loss patients who received pretreatment with estradiol valerate and/or mifepristone, followed by carboprost methylate suppositories (study groups), or carboprost methylate suppositories alone (control group) in a regional central institution from March 2020 to February 2021 were retrospectively studied. All patients were evaluated by hysteroscopy 6 h after carboprost methylate suppositories use to assess whether the gestational sac was complete expulsion and assess the morphology of uterine cavity.

**Results:**

The complete expulsion rate was 56.94% in the mifepristone and estradiol valerate-pretreatment group, 20.69% in the estradiol valerate-pretreatment group, 62.5% in the mifepristone-pretreatment group, and 12.5% in the control group. Compared with the control group, pretreatment with estradiol valerate did not increase the complete expulsion rate significantly (*P* = 0.297), pretreatment with mifepristone increased the complete expulsion rate significantly (*P* < 0.001). Pretreatment with mifepristone combined with estradiol valerate did not increase the complete expulsion rate significantly comparing with pretreatment with mifepristone (*P* = 0.222). The data of IUA showed that the complete expulsion rate in patients with IUA was lower than that in those patients without IUA (*P* < 0.001).

**Conclusions:**

Pretreatment with estrogen was not a sensible substitute for mifepristone in the medical management of early pregnancy loss. Mifepristone followed by carboprost methylate suppositories was likelihood of the ideal medical scheme in early pregnancy loss. IUA decreased the complete expulsion rate of medical management, it is cautious about medical management for early pregnancy loss with risk of IUA.

*Trial Registration* Number: ChiCTR2100046503. Date of registration (retrospectively registered): May 18, 2021. Trial registration website: http://www.chictr.org.cn/.

## Introduction

Early pregnancy loss is the most common complication in the first trimester, including anembryonic gestation and embryonic or fetal death [1]. Some patients have symptoms, such as vaginal bleeding and uterine cramping. Accepted treatment options for early pregnancy loss include expectant, medical and surgical management [1]. Although the reported success rate of expectant management was 43% ~ 80% [1–4], patients generally prefer medical management because of a long restless time [5, 6]. The success rate of standard medical management with misoprostol alone ranged from 40 to 87% [2, 6–9]. Mifepristone pretreatment with misoprostol was recommended in early pregnancy medication abortion [10, 11]. However, the embryo development has stopped in early pregnancy loss, and there is some doubt as to whether mifepristone competitive binding to progesterone receptor can increase the success rate of complete expulsion or not. The empirical addition of estrogen pretreatment is common in clinic of East Asia. However, there was little reported the effect of estrogen in the medical management of early pregnancy loss. The optimal schedule of medical management for early pregnancy loss needs to be determined urgently. In addition, intrauterine adhesion (IUA), as a non-negligible uterine factor, should be considered for the incidence of early pregnancy loss [12], which is likely to be the crucial factor influencing the effect of medical management, but clinical evidence is limited.

This study aimed to report our experience with current drugs for medical management of early pregnancy loss and describe the relationship between IUA and efficacy of medical management, which further provides a recommendation for optimal medical scheme of early pregnancy loss.

## Materials and methods

### Ethical approval

Ethics approval for the study was granted by the Ethics Committee of the first affiliated Hospital of Chongqing Medical University (approval number 058/2020) to undertake a retrospective study for cases with early pregnancy loss treated by medication combined with hysteroscopy from March 2020 to February 2021 in this Regional Medical Centre. The informed consent was taken from all individual participants.

### Study design

Healthy adult women were eligible if they had been diagnosed with a nonviable intrauterine pregnancy through vaginal ultrasound examination between 6 and 12 completed weeks of gestation. The exclusion criteria included cornual pregnancy, cesarean scar pregnancy, and cervical pregnancy. Women in whom mifepristone, estradiol valerate or carboprost methylate suppositories were contraindicated were also excluded. Patients with complete or incomplete pregnancy tissue expulsion before the use of carboprost methylate suppositories were still excluded. Only cases who completed the procedure including carboprost methylate suppositories vaginally were included in this study. All 226 participants completed the trial. The participants received therapeutic schedule as follows: (1) estradiol valerate 5 mg bid × 3 days and mifepristone (morning 50 mg, evening 25 mg) × 2 days orally followed by 1 mg carboprost methylate suppositories vaginally (mifepristone and estradiol valerate-pretreatment group) 24 h later, (2) estradiol valerate 5 mg bid × 3 days orally followed by 1 mg carboprost methylate suppositories vaginally (estradiol valerate-pretreatment group) 24 h later, (3) mifepristone (morning 50 mg, evening 25 mg) × 2 days orally followed by 1 mg carboprost methylate suppositories vaginally (mifepristone-pretreatment group) 24 h later, (4) 2 days waiting followed 24 h by 1 mg carboprost methylate suppositories vaginally alone (methylate carboprost suppositories-alone group).

All participants were treated with carboprost methylate suppositories, and then were examined by hysteroscopy to evaluate complete expulsion and morphology of uterine cavity. Complete expulsion referred to the absence of gestational sac in uterine cavity. If residue existed, uterine aspiration was performed under hysteroscopy.

Calculate the success rate of complete expulsion and incidence of IUA, analyze the relationship between IUA and medical management results, and record the side effects of medical management after use of carboprost methylate suppositories.

### Statistical analyses

Data analyses were performed with SAS9.14. All patients were provided informed consent before they were involved in the study. We calculated the percentage (with 95% confidence interval) of women in each group who had complete pregnancy tissue expulsion and compared the results using Chi-squared Test. The optimal combination drugs, the relationship between IUA and efficacy of medical management of early pregnancy loss were analyzed by Logistic regression model.

## Results

**Fig. 1:**
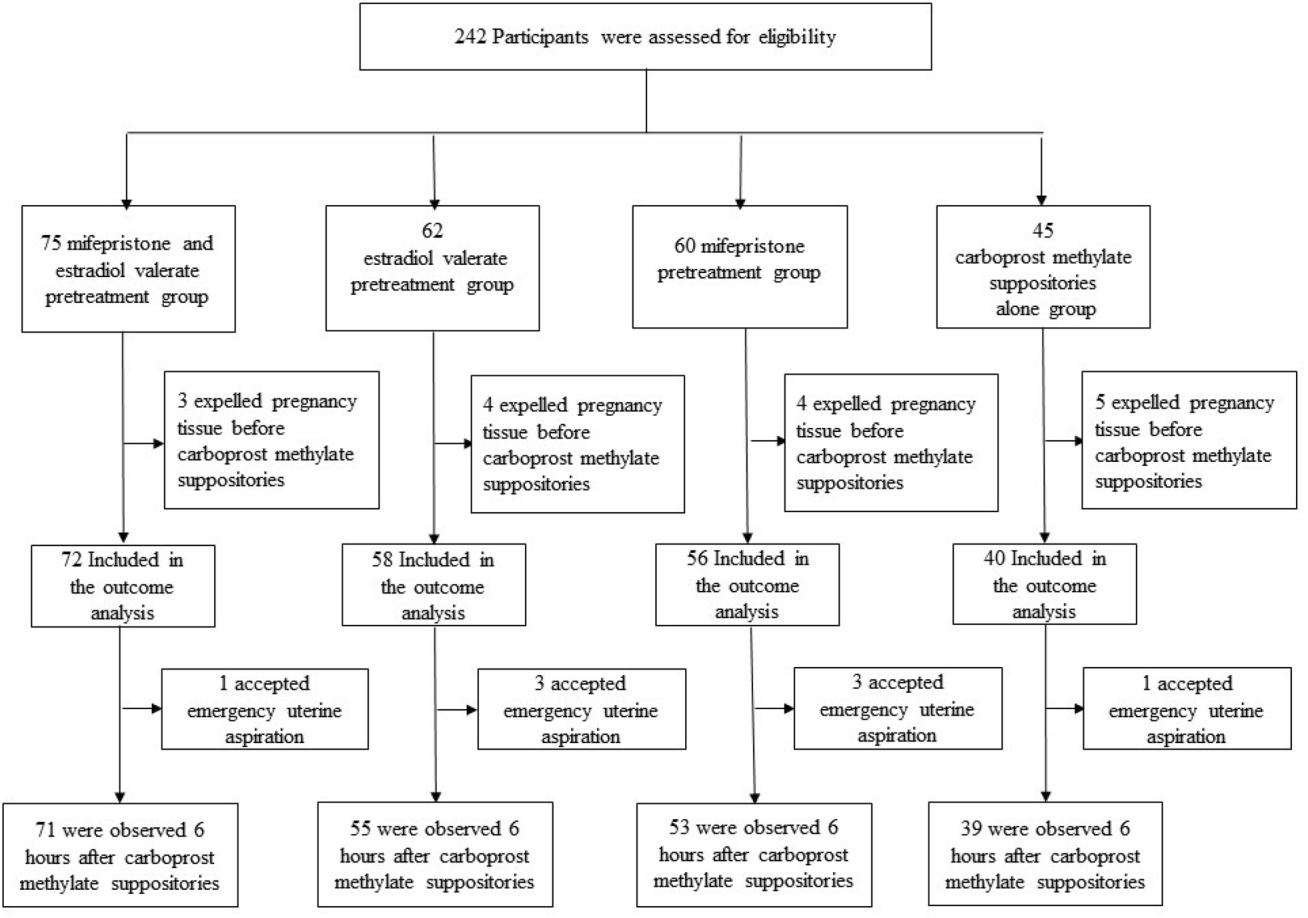
Enrollment, exclusion, follow-up, and analysis.

From March 2020 to February 2021, we assessed 242 women for eligibility. Other 16 patients were excluded, because they expelled pregnancy tissue completely or incompletely before carboprost methylate suppositories used vaginally. 226 patients were included in the study (Fig. 1).

There were 8 cases (1 patient in mifepristone and estradiol valerate-pretreatment group, 3 patients in estradiol valerate-pretreatment group, 3 patients in mifepristone pretreatment group, and 1 patient in carboprost methylate suppositories-alone group) accepted emergency uterine aspiration followed by hysteroscopy due to massive bleeding after 1 mg carboprost methylate suppositories less than 6 h, these cases finished the scheme identified as failed treatment. 218 participants were observed 6 h after management with carboprost methylate suppositories, and then were examined by hysteroscopy to evaluate complete expulsion and morphology of uterine cavity (Fig. 1).

**Fig. 2:**
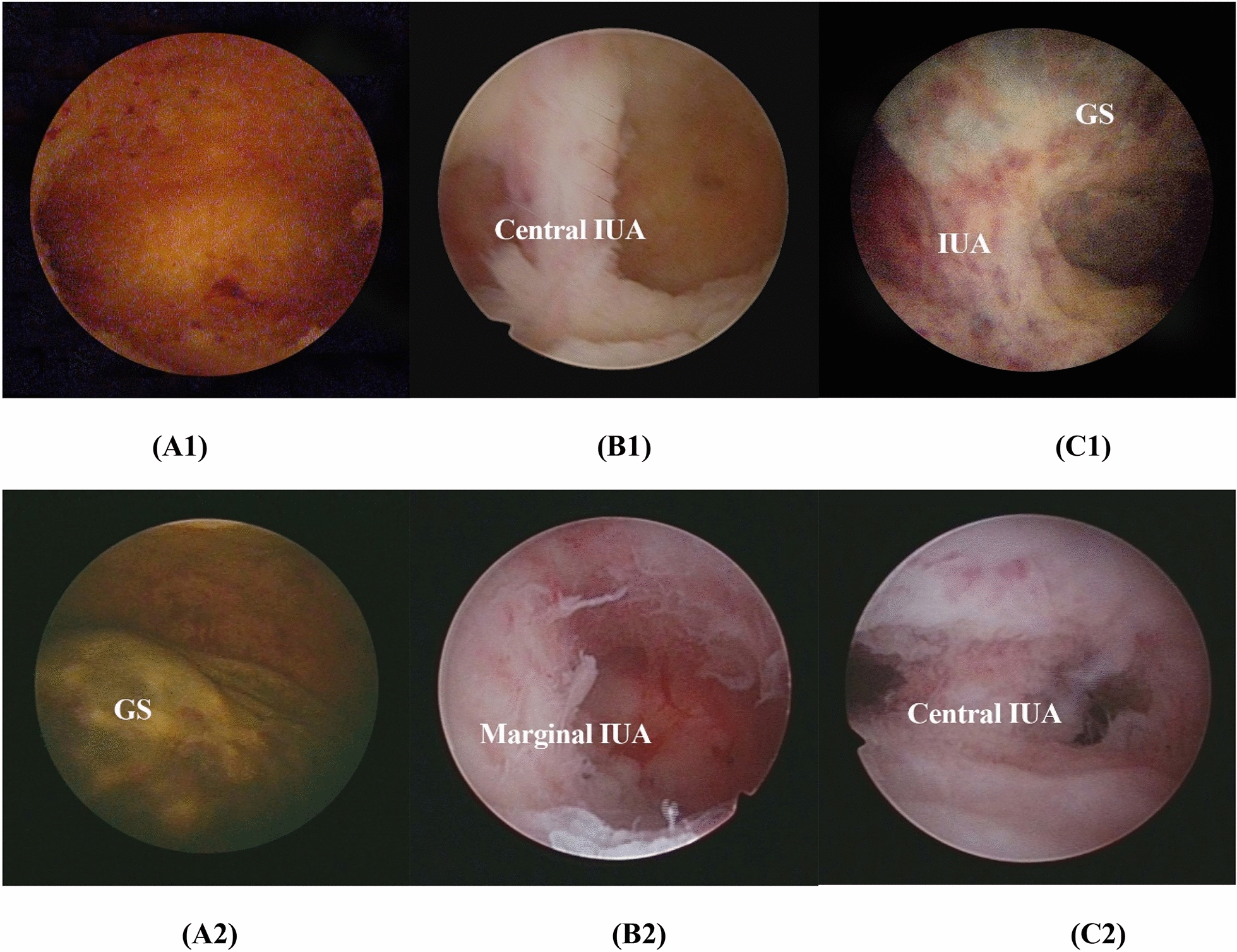
Hysteroscopy visualization after medical management 6 h.

The intrauterine manifestations after medical management and the morphology of uterine cavity after uterine aspiration could be visually evaluated by hysteroscopy. If complete expulsion occurred, there was no sac in the uterine cavity (Fig. 2A1); instead, the sac was still in the uterine cavity (Fig. 2A2), then uterine aspiration should be performed under hysteroscopy. The band of scar adhesion tissue was found in the middle of the uterine cavity (central IUA) (Fig. 2B1), and the scar adhesion tissue was found on the wall of the uterine cavity (marginal IUA) (Fig. 2B2) after uterine aspiration. We even found both gestational sac and adhesion band in the uterine cavity (Fig. 2C1), and then band of scar adhesion tissue was observed in the middle of the uterine cavity when the same patient finished uterine aspiration (Fig. 2C2).

### Compare the complete expulsion rate of different groups

Analyses of the primary outcome according to patient demographics and clinical characteristics were prespecified; we performed analyses that were stratified according to maternal age, gestational weeks, previous loss pregnancy times, previous induced abortion times, mean sac diameter (MSD) and parity. The number of cases with IUA diagnosed by hysteroscopy was also no significant difference in each group. Baseline characteristics were similar in the four treatment groups (Table 1).

**Table 1:** Characteristics of the participants at baseline*

Project	Mifepristone and estradiol valerate-pretreatment group (*N* = 72)	Estradiol valerate-pretreatment group (*N* = 58)	Mifepristone-pretreatment group (*N* = 56)	Methylate carboprost suppository-alone group (*N* = 40)	*P*
Maternal age, mean ± SD	31.63 ± 4.32	30.91 ± 5.52	30.98 ± 5.41	31.13 ± 3.82	0.8316
Gestational weeks, mean ± SD	9.85 ± 1.25	9.81 ± 1.54	9.82 ± 1.62	9.88 ± 1.34	0.9943
Previous early pregnancy loss times, mean ± SD	0.57 ± 0.85	0.45 ± 0.82	0.29 ± 0.56	0.25 ± 0.44	0.1182
Previous induced abortion tines, mean ± SD	0.6 ± 0.78	0.76 ± 0.84	0.54 ± 0.85	0.4 ± 0.67	0.0784
MSD, mean ± SD	24.15 ± 9.33	25.82 ± 9.42	22.17 ± 11.29	24.74 ± 8.89	0.2516
Parity, mean ± SD	0.39 ± 0.52	0.4 ± 0.56	0.61 ± 0.68	0.3 ± 0.52	0.0895
IUA, n(%)	28 (38.89%)	20 (34.48%)	14 (25.00%)	9 (22.50%)	0.198

IUA: Intrauterine adhesion; MSD: Mean sac diameter; SD: standard deviation

*Plus–minus values are means ± SD. There were no significant differences between the groups in any of the characteristics listed. Percentages may not sum to 100 because of rounding

Complete expulsion 6 h after carboprost methylate suppositories occurred in 41 of 72 women (56.94%; 95% confidence interval [CI] 44.51% to 68.38%) in the mifepristone and estradiol valerate-pretreatment group, in 12 of 58 women (20.69%; 95% CI 10.26% to 31.11%) in the estradiol valerate-pretreatment group, in 35 of 56 women (62.5%; 95% CI 49.82% to 75.18%) in the mifepristone-pretreatment group, and in 5 of 40 women (12.5%; 95% CI 2.25% to 22.75%) in the carboprost methylate suppositories-alone group (absolute difference in the rate of treatment success, $\chi^{2}$ = 41.54, *P* < 0.001) (Table 2). By Logistic regression analysis for pretreatment factors, mifepristone had significant effect on the success rate of complete expulsion (*P* < 0.001), estradiol valerate had no significant effect on the success rate of complete expulsion (*P* = 0.297), mifepristone combined with estradiol valerate had no significant effect comparing with mifepristone either (*P* = 0.222) (Table 3).

**Table 2:** Success complete expulsion rate in each group

Outcome	mifepristone and estradiol valerate-pretreatment group (*N* = 72)	estradiol valerate-pretreatment group (*N* = 58)	Mifepristone-pretreatment group (*N* = 56)	methylate carboprost suppository-alone group (*N* = 40)	Statistic	*P*
	Number(percent)				*Χ*^2^ = 41.54	< 0.0001
Gestational sac complete expulsion: treatment success	41	12	35	5		
	56.94%	20.69%	62.5%	12.5%		
95% CI	44.51%, 68.38%	10.26%, 31.11%	49.82%, 75.18%	2.25%, 22.75%

**Table 3:** Efficacy of mifepristone with/or estradiol valerate pretreatment

Parameter	Regression coefficient	Standard error	Wald Chi-square	*P* value	Parameter	OR	95% CI	
Intercept	− 1.9459	0.4781	16.5662	< .0001	Estradiol valerate at Mifepristone = not use	1.826	0.589	5.665
Estradiol valerate	0.6022	0.5776	1.0868	0.2972	Estradiol valerate at Mifepristone = use	0.794	0.388	1.621
Mifepristone	2.4567	0.5521	19.8041	< .0001	Mifepristone at Estradiol valerate = not use	11.667	3.954	34.423
Estradiol valerate*mifepristone	− 0.8334	0.683	1.489	0.2224	Mifepristone at Estradiol valerate = use	5.07	2.305	11.151

### Analyze the influence from IUA on the results of medical management

All patients after carboprost methylate suppositories vaginally were examined by hysteroscopy, and it was found that IUAs were as follows: 28 of 72 women (38.89%; 9 in success, 19 in failure) in the mifepristone and estradiol valerate-pretreatment group, 20 of 58 women (34.48%; 3 in success, 17 in failure) in the estradiol valerate-pretreatment group, 14 of 56 women (25%; 4 in success, 10 in failure) in the mifepristone-pretreatment group, and 9 of 40 women (22.5%; 0 in success, 9 in failure) in the carboprost methylate suppositories-alone group. Logistic regression analysis showed the rate of complete expulsion in patients with IUA was lower significantly than that in those without IUA (*P* < 0.001; OR: 0.20; 95% CI 0.098 to 0.415) (Table 4).

**Table 4:** Influence of IUA on the outcome of medical management

Parameter		Estimate	Standard Error	Wald Chi-Square	Pr > ChiSq	OR	95% CI	
Intercept		− 1.7252	0.484	12.7026	0.0004			
IUA	Yes	− 1.5997	0.3677	18.9289	< .0001	0.202	0.098	0.415
Group	Mifepristone and estradiol valerate-pretreatment	2.6531	0.5614	22.3318	< .0001	14.198	4.724	42.669
Group	Estradiol valerate-pretreatment	0.77	0.5883	1.7132	0.1906	2.16	0.682	6.841
Group	Mifepristone-pretreatment	2.6674	0.5702	21.8871	< .0001	14.403	4.711	44.033

IUA: Intrauterine adhesion

### Side effects and follow-up

The adverse events for all participants were as follows: 8 (3.54%) patients experienced massive vaginal bleeding, 28 (12.39%) patients experienced nausea, 23 (10.18%) patients experienced vomiting, 15 (6.64%) patients experienced diarrhea and only 1 (0.44%) patient experienced fever.

All specimens were sent for pathological examination, and no obvious abnormality was found. We followed up all participants by telephone and menstruation of all participants was recovered with no residual pregnancy tissue at the end of follow-up.

## Discussion

In this retrospective study, pretreatment with mifepristone had significantly increased the rate of complete expulsion, while the additional use of estradiol valerate did not improve the rate of complete expulsion significantly. Pretreatment with estrogen was not a sensible substitute for mifepristone. Mifepristone combined with carboprost methylate suppositories should be recommended for medical management of early pregnancy loss. IUA decreased the success rate of complete expulsion significantly. IUA, as a risk factor of morbidly adherent placentation or placenta implantation [13], can block the passage to expel the pregnancy sac. In view of the relatively low success rate of complete expulsion, it is necessary to consider uterine aspiration under hysteroscopy for patients with risk factors of IUA, such as the history of recurrent miscarriages, more than 2 times of dilation and curettage, multiple pregnancies, retained products of conception and placenta implantation [14, 15].

Estradiol valerate [16] can increase the sensitivity of the uterus to prostaglandin and promote pregnancy sac expulsion in theory. Our study showed that empirical pretreatment with estrogen did not increase the complete expulsion rate in early pregnancy loss. However, the mechanism remains to be further studied.

Carboprost methylate suppositories is a non-injectable derivative of prostaglandin (PG) F2α, which is similar to the injectable carboprost that is widely used in refractory uterine atony [17, 18]. This medication with significant excitatory uterine smooth muscle has been recommended for use in medication abortion combined with mifepristone in China [19]. It suggested that with a dose of 1 mg, the effective rate of medication abortion in early pregnancy is 95.2–95.7% [20]. It is similar to the protocol of mifepristone combined with misoprostol (95–99%) which is recommended by the United States guideline [10, 11, 21, 22]. On the other hand, the common adverse effects of misoprostol, such as nausea, abdominal pain, vomiting, diarrhea, and fever, occurred in more than 50% of women [11, 22]. While the adverse effects incidence of carboprost methylate suppositories occurred in 34.5% of women according to the limited literatures [23]. What's more, the rate of side effects in our study was 33.19%, which was lower than that reported in the literature. Therefore, carboprost methylate suppositories 1 mg were used vaginally in our study. Nevertheless, carboprost methylate suppositories is not equated with misoprostol for medical management of early pregnancy loss, and its efficacy remains to be further studied. We are going to do more research on the use of carboprost methylate suppositories in pharmacology and clinic.

Hysteroscopy is the gold standard for the diagnosis of IUA [24, 25]. The diagnosis of IUA in early pregnancy by hysteroscopy has been rarely reported. The uterine cavity is dilated and deformed in pregnancy. Those with obvious central adhesions are easily diagnosed subjectively, and it is difficult to diagnose the marginal type of adhesions. Although all hysteroscopies in this study were performed by one senior physician to minimize the bias, the actual incidence of IUA is perhaps more than our data. Therefore, the diagnosis of IUA at the time of abortion is not the fantastic opportunity. It may be better to evaluate the morphology of uterine cavity by hysteroscopy until uterus is complete involution.

## Data Availability

Data and other materials can be made available by the corresponding author upon a reasonable request.

## Abbreviations

IUA: Intrauterine adhesion
MSD: Mean sac diameter
